# Tissue-specific alternative splicing separates the catalytic and cell signaling functions of human leucyl-tRNA synthetase

**DOI:** 10.1016/j.jbc.2022.101757

**Published:** 2022-02-21

**Authors:** Max Baymiller, Benjamin Nordick, Connor M. Forsyth, Susan A. Martinis

**Affiliations:** Department of Biochemistry, University of Illinois Urbana-Champaign, Urbana, Illinois, USA

**Keywords:** aminoacyl-tRNA synthetase, alternative splicing, transfer RNA (tRNA), translation, mammalian target of rapamycin (mTOR), serine/arginine-rich splicing factor 1 (SRSF1), AARS, aminoacyl-tRNA synthetases, EPRS, glutamyl-prolyl tRNA synthetase, IFN-γ, interferon gamma, LARS, leucyl-tRNA synthetase, LSV, LARS splice variant, MSC, multisynthetase complex, mTOR, mammalian target of rapamycin, PMA, phorbol 12-myristate 13-acetate, RBP, RNA-binding protein, SRSF1, serine-arginine-rich splicing factor 1, TCA, trichloroacetic acid, UIUC, University of Illinois Urbana-Champaign

## Abstract

The aminoacyl-tRNA synthetases are an ancient and ubiquitous component of all life. Many eukaryotic synthetases balance their essential function, preparing aminoacyl-tRNA for use in mRNA translation, with diverse roles in cell signaling. Herein, we use long-read sequencing to discover a leukocyte-specific exon skipping event in human leucyl-tRNA synthetase (LARS). We show that this highly expressed splice variant, LSV3, is regulated by serine-arginine-rich splicing factor 1 (SRSF1) in a cell-type-specific manner. LSV3 has a 71 amino acid deletion in the catalytic domain and lacks any tRNA leucylation activity *in vitro*. However, we demonstrate that this LARS splice variant retains its role as a leucine sensor and signal transducer for the proliferation-promoting mTOR kinase. This is despite the exon deletion in LSV3 including a portion of the previously mapped Vps34-binding domain used for one of two distinct pathways from LARS to mTOR. In conclusion, alternative splicing of LARS has separated the ancient catalytic activity of this housekeeping enzyme from its more recent evolutionary role in cell signaling, providing an opportunity for functional specificity in human immune cells.

Aminoacyl-tRNA synthetases (AARS) are ancient, ubiquitous, and essential enzymes, which attach amino acids to their cognate tRNAs (1). In doing so, AARS set the genetic code and determine much of the fidelity of translation (2). The AARS have also accrued a multitude of functions that are entirely separate from their canonical tRNA aminoacylation activity (3). In humans, these include AARS regulating events as disparate as angiogenesis (4) and apoptosis (5). Idiosyncratic AARS-dependent regulation occurs through diverse mechanisms in transcription (6), splicing (7), translation (8) and cell–cell signaling (9).

Modifications are often used to divert AARS proteins from their essential enzymatic roles to alternative functions. For example, glutamyl-prolyl tRNA synthetase (EPRS) is phosphorylated during interferon gamma (IFN-γ) signaling, preventing interaction with the multi-AARS complex (MSC) and allowing it to facilitate antiviral responses by binding specific mRNAs and inhibiting their translation (8, 10, 11). Phosphorylation of lysyl-tRNA synthetase (KARS) during allergen exposure also leads to its release from the MSC and induces a conformational change, which inhibits KARS translational activity while enhancing its orthogonal function in transcriptional activation of immunity genes (6, 12).

In other cases, removal of a large portion of the AARS protein is required to control noncanonical activities. Tyrosyl-tRNA synthetase (YARS) is proteolytically processed to separate the main catalytic protein body from its 169 amino acid EMAP II cytokine-like domain (13). The resulting YARS fragments are then secreted and have distinct extracellular signaling activities (9, 14). Beyond these posttranslational modifications, the mRNA-binding activity of EPRS can be modulated *via* polyadenylation of the coding region of the EPRS transcript, generating a C-terminally truncated “EPRS^N1^” protein, which relieves translation inhibition caused by the full-length protein (15).

Alternative splicing has also been shown to regulate AARS and their noncanonical function by removing large portions of the coding sequence. During IFN-γ signaling, tryptophanyl-tRNA synthetase (WARS) undergoes an exon skipping event to remove a 47 amino acid portion of its N-terminus (16, 17). The resultant “mini” WARS is a potent inhibitor of angiogenesis (4). Alternative splicing of histidyl-tRNA synthetase (HARS) and YARS is also known to generate novel structural conformations not present on the native proteins (18, 19). Hundreds of further alternative splice variants (SVs) of AARS have been discovered in humans and mice, most of which disrupt the coding sequence of the essential and conserved aminoacylation domains (20), but their functions are still unknown.

Human cytoplasmic leucyl-tRNA synthetase (LARS) is a large multidomain AARS with a crucial moonlighting role as an intracellular leucine sensor (21). Upon leucine binding, LARS activates mammalian target of rapamycin (mTOR), a master regulator of cell growth and translation, through two distinct pathways involving RagD GTPase (21) and Vps34 lipid kinase (22). While leucine sensing does not require catalysis (23), both activities are coordinately inhibited by phosphorylation of LARS during glucose starvation (24). No mechanism is currently known for separately regulating either the canonical enzymatic or alternative cell signaling functions of LARS without influencing the other.

Multiple alternative splicing events occur on *LARS* mRNA in humans (20). We hypothesized that these may lead to SV isoforms that selectively disrupt either the catalytic or leucine sensing roles of LARS, creating functionally specialized proteins for use in specific cellular contexts. Therefore, we set out to identify alternatively spliced *LARS* transcripts with promise for novel biology.

## Results

### Long-read sequencing identifies a highly expressed, tissue-specific LARS splice variant

Previous reports showed that immunological tissues contain a large number of AARS SVs (20). This observation, coupled with the frequent involvement of AARS in immune biology (6, 8, 14, 17), led us to target primary leukocytes as a diverse and promising tissue source from which to discover LARS splice variants (LSVs) *via* deep sequencing.

The full-length *LARS* mRNA is comprised of 33 coding exons across 3.9 kb. To comprehensively identify alternative splicing events across the long and complex *LARS* transcript, we employed a strategy that included Pacific Biosciences (PacBio) sequencing. The PacBio approach generates exceptionally long reads (up to 15 kb), allowing for detection of multiple nonadjacent alternative splicing events on the same transcript. The depth of sequencing also allows for detection of especially rare isoforms (25).

**Figure 1:**
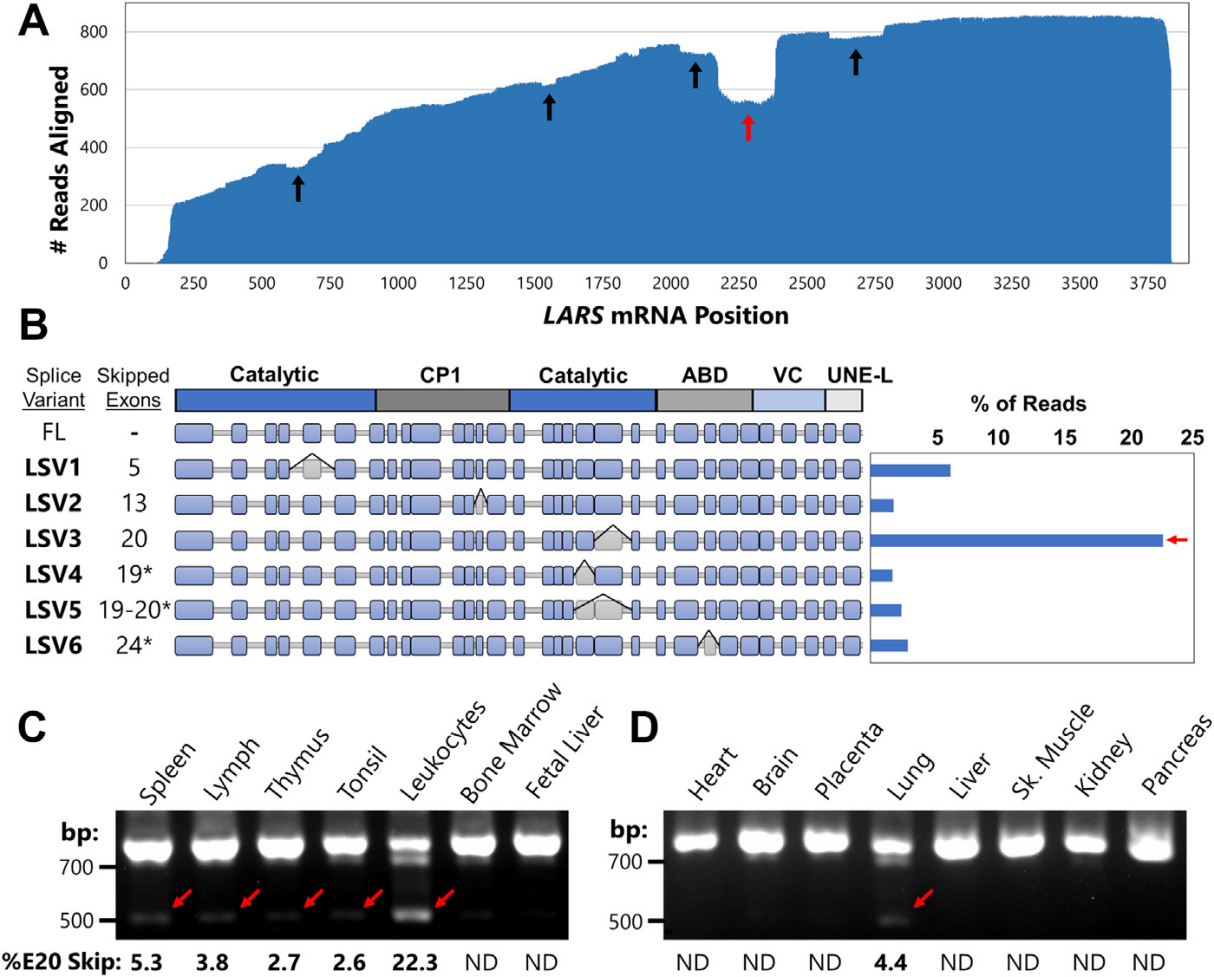
**LSV3 is a highly expressed and leukocyte-specific splice variant of LARS.***A*, base presence graph showing alignment of high-quality reads from Pacific Biosciences sequencing of leukocyte polyA+ RNA to the *LARS* mRNA sequence. The *arrows* indicate exon-skipping events, with several overlapping events indicated by the *red arrow*. *B*, quantification of PacBio results. *Top line diagram* represents the protein structure, with LARS splice variants (LSV) 1 to 6 shown on the exon structure of *LARS* mRNA. *Asterisks* indicate exon skipping-induced frameshifts. *C*, agarose gel of RT-PCR using primers spanning a 721 bp region of the *LARS* transcript, on cDNA from human immune tissues. *Lower band* indicates skipping of the 213 bp exon 20 (E20) in LSV3. *D*, as in *C*, but with cDNA from eight diverse human tissues. LARS, leucyl-tRNA synthetase.

A *LARS* gene-specific cDNA library was constructed from leukocyte polyA+ RNA (Takara Biosciences). Sequencing *via* PacBio of these *LARS* mRNAs in leukocytes resulted in approximately 11,000 sequencing reads with an average length of 2.7 kb (Fig. S1*A*). Alignment of high-quality reads with the *LARS* reference mRNA revealed several distinct exon skipping events (Fig. 1*A*). The extensive length of the PacBio reads allowed us to conclude that exon skipping events did not co-occur with any other alternative splicing features on the same transcripts (Fig. S1*B*). These six *LARS* transcript isoforms were thus denoted LSV 1 to 6 (Fig. 1*B*). The LSV1-3 and LSV5 isoforms have been previously reported (P. Schimmel, personal communication) (20), while LSV4 and LSV6 are newly discovered.

Strikingly, LSV3, which skips exon 20, dominated the alternative splicing landscape of LARS in leukocytes, representing over 20% of reads in the PacBio dataset (Fig. 1*B*). Because of its relatively high expression, we wondered if LSV3 was present in other biological contexts. We used PCR to amplify cDNA from a spectrum of human tissues with primers flanking a 721 bp portion of the *LARS* gene transcript surrounding exon 20 and separated the products by electrophoresis. In leukocytes, we detected not only the full-length *LARS* transcript, but also a 508 bp product that would correspond to a spliced transcript that skips the 213 bp exon 20 (Fig. 1*C*). A second ∼690 bp band detected in leukocytes did not match any known LSVs from our PacBio dataset or exon sizes in this region. Quantification of the LSV3 band in leukocytes reflected 22% exon 20 skipping, in excellent agreement with the PacBio sequencing results described above.

Exon 20 skipping was also detected at low levels (5% or less) in most human immune tissues that we tested, including the spleen and thymus (Fig. 1*C*). The only exceptions were tissues originating from immature immune progenitor cells, such as the bone marrow and fetal liver. In contrast, no LSV3 was detected in major nonimmune tissues such as the heart, brain, liver, and muscle (Fig. 1*D*). The only nonimmune system tissue that showed a small amount of exon 20 skipping was the lung. We hypothesized that this might be due to the presence of alveolar macrophages or other resident immune cells, which are abundant in the lung. These data collectively suggest that LSV3 is a highly leukocyte-specific alternative SV of LARS.

### LSV3 splice variant mRNA codes for a catalytically inactive LARS protein

Human LARS is a large protein, comprised of 1176 amino acids with multiple distinct functional domains. These include the class I AARS Rossmann fold catalytic domain (26) containing the canonical aminoacylation active site, the dedicated tRNA editing domain (CP1; (27)), so-called anticodon binding (ABD) and variable C-terminus (VC) domains, which are involved in tRNA binding (28), and the UNE-L domain, which binds LARS to a multisynthetase complex (MSC) (29).

**Figure 2:**
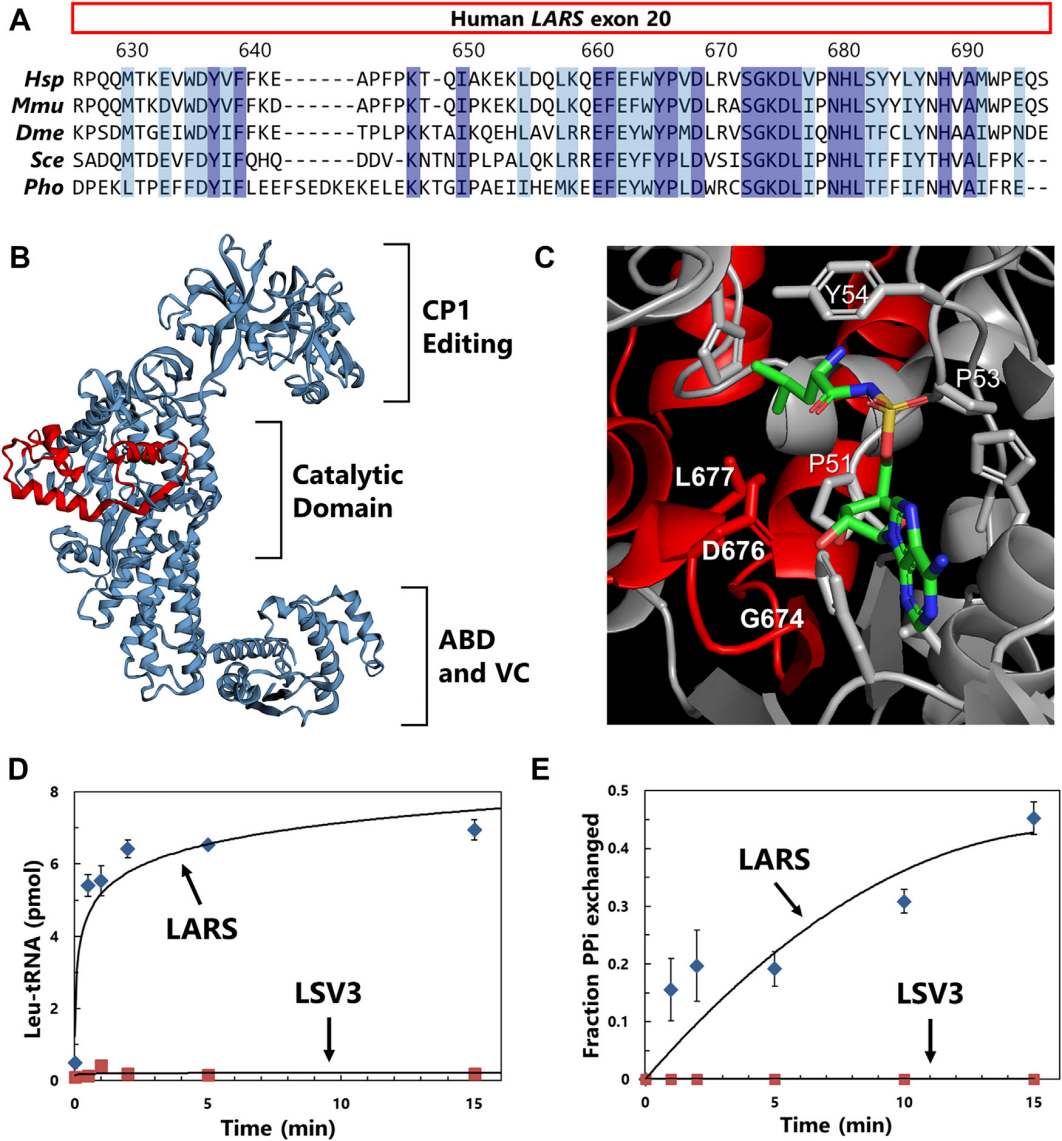
**Exon 20 skipping creates a catalytic null LARS.***A*, alignment of human LARS exon 20 protein sequence with diverse eukaryotic and archaeal LARS. Fully conserved residues colored *blue*, highly similar residues in *light blue*. Hsp = *Homo sapiens*, Mmu = *Mus musculus*, Dme = *Drosophila melanogaster*, Sce = *Saccharomyces cerevisiae*, Pho = *Pyrococcus horikoshii*. *B*, structure of human LARS (PDB 6LPF) with the region encoded by exon 20 colored *red*. *C*, enlarged view of LARS active site showing key exon 20 residues (*red ribbon*, *bold labels*) interacting with the leucyl-adenylate intermediate. *D*, aminoacylation assay of LARS and LSV3 conducted using 10 μC [^3^H]-leucine, 2 mg/ml bovine total tRNA, and 50 nM of recombinant enzyme. All reactions were run in triplicate. *E*, pyrophosphate exchange assay of LARS and LSV3 using 0.025 μCi [^32^P]-pyrophosphate, 4 mM ATP, and 50 nM recombinant enzyme. ATP and PP_*i*_ were separated by TLC and quantified. LARS, leucyl-tRNA synthetase.

Deletion of exon 20 does not disrupt the reading frame of LARS, but does eliminate 71 amino acids in the catalytic domain. This portion of the LARS sequence is well conserved and contains a number of residues, which are invariant across a wide selection of eukaryotes and even archaea (Fig. 2*A*). Mapping these residues onto the structure of human LARS (28) (Fig. 2*B*) shows that they include an α-helix and β-strand in the Rossman fold catalytic core, as well as a helix-turn-helix in the variable leucine-specific domain 2 (LSD2), the function of which is unknown. The amino acids flanking the deletion are about 30 Å apart in the structure. Several of the most conserved residues in exon 20 likely participate in ATP binding and activation during intermediate steps of catalysis (28) (Fig. 2*C*, bold). Furthermore, it is possible that tRNA binding would be disrupted by this deletion, as the LARS catalytic domain interacts with the tRNA acceptor stem (30). Therefore, we would predict that LSV3 is catalytically inactive.

We recombinantly expressed LSV3 fused to an N-terminal six-histidine tag in *E. coli*. The His-tagged protein was isolated *via* nickel-affinity chromatography as soluble protein (Fig. S2*A*). As would be expected from the primary sequence, we found that LSV3 did not aminoacylate crude tRNA with leucine (Fig. 2*D*) under conditions in which recombinant full-length human LARS was active. We also determined that the LSV3 protein could not activate leucine to form an adenylate in the first step of aminoacylation (Figs. 2*E* and S2*B*). Thus, deletion of exon 20 clearly disrupts the aminoacylation catalytic activity of the LARS housekeeping protein.

### LSV3 is a functional leucine-sensing protein for the mTOR signaling pathway

In addition to its essential function in tRNA leucylation, human LARS serves as an intracellular leucine sensor for the mTOR pathway (21) which controls many aspects of cell growth. In this capacity, LARS binds leucine using the same site as in catalysis and undergoes a conformational change that enhances its interaction with RagD GTPase (31). Through a largely undefined mechanism, LARS facilitates the bound RagD to perform GTP hydrolysis to GDP (23). This GDP-RagD then promotes mTOR complex 1 (mTORC1) kinase activity and downstream signaling (32). LARS also binds to and activates Vps34 lipid kinase, which positively signals to mTORC1 through an orthogonal pathway (22).

**Figure 3:**
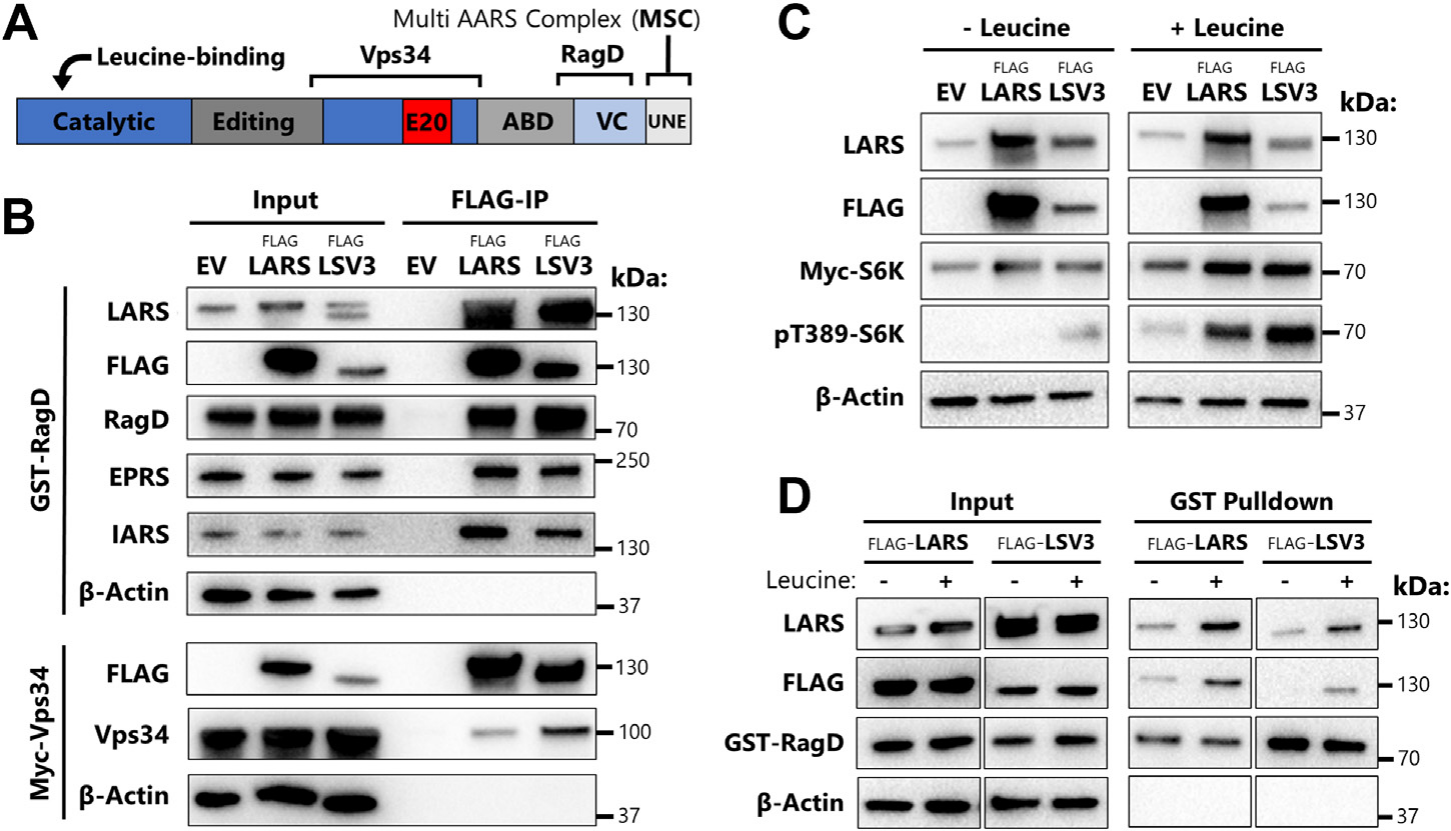
**LSV3 retains functionality as a leucine sensor for mTOR.***A*, *line diagram* showing sites on LARS protein involved in mTOR signaling. *B*, Western blot of FLAG-tag immunoprecipitation (IP) from 293T cells transfected with pcDNA3 empty vector (EV), FLAG-LARS or LSV3 and GST-RagD or Myc-Vps34. All IP’s were performed at least in duplicate. EPRS = glutamyl-prolyl-tRNA synthetase, IARS = Isoleucyl-tRNA synthetase. *C*, Western blot of 293T cells transfected with EV, FLAG-LARS or LSV3 and Myc-S6K. Cells were starved of serum for 20 h, followed by 2 h in leucine-free DMEM (−Leucine) and then 20 min with 4 mM leucine (+Leucine). Ratio of pT389-S6K to Myc-S6K was quantified (n = 4). *D*, 293T cells were transfected with FLAG-LARS or LSV3 and GST-RagD, leucine starved or stimulated as in *C*, and then subjected to GST tag pull-down with glutathione resin. LARS, leucyl-tRNA synthetase; mTOR, mammalian target of rapamycin.

Despite removal of a portion of the catalytic domain for aminoacylation, LSV3 retains key features in the primary sequence that are important for mTOR signaling, including the conserved leucine-binding site and RagD-binding peptide (21) (Fig. 3*A*). Interestingly, exon 20 is contained within the large region identified for Vps34 binding (22). The presence of the idiosyncratic UNE-L domain also suggests that LSV3 would retain binding to the MSC that is comprised of eight AARS and three nonsynthetase proteins (29).

To investigate LSV3’s protein–protein interactions, we modified a vector encoding an N-terminal FLAG epitope tag-fused LARS gene to delete bases for exon 20 and expressed the recombinant protein (FLAG-LSV3) in HEK 293T cells. Coimmunoprecipitation (co-IP) using the FLAG tag showed that, similar to LARS, LSV3 interacts with isoleucyl- and glutamyl-prolyl-tRNA synthetases (IARS and EPRS, Fig. 3*B*), supporting that this SV is indeed a part of the MSC. We also found that LSV3 interacts with the PHD1 oxygen sensor, a reported regulator of LARS protein stability (33) (Fig. S3*A*).

Interestingly, co-IP demonstrated that LSV3 maintains interactions with downstream mTORC1 signaling partners. As expected from the presence of the C-terminal RagD-binding site in the SV sequence (Fig. 3*B*), LSV3 interacts with the mTOR signaling partner RagD GTPase. More surprisingly, co-IP using FLAG-LSV3 also pulled down Myc-Vps34 (Fig. 3*B* bottom panel). This is despite apparent disruption to the previously identified Vps34 binding site (22) (Fig. 3*A*). Reciprocal IP of the Myc-Vps34 confirmed the interaction between Vps34 and LSV3 (Fig. S3*B*). While the residues deleted in the LSV3 SV are within the known Vps34-binding site, this region was identified using a 360 amino acid fragment of LARS (22). Thus, our results with this SV suggest that the location of the Vps34-binding site can be further narrowed to the protein surface on the LARS catalytic and CP1 domains that are formed by residues 360 to 625 (Fig. S3*C*).

Previous work has established that leucine sensing by LARS is distinct from, and does not require, its tRNA leucylation activity (21, 22, 23). While protein interactions with downstream partners RagD and Vps34 suggest that LSV3 maintains its role in mTORC1 signaling, it is possible that the significant disruption caused by skipping of the 71 amino acids encoded by exon 20 has abolished this function. We directly tested mTORC1 signaling by the SV in HEK 293T cells expressing either LSV3 or LARS. Cells were starved of leucine and serum, followed by brief leucine stimulation, and mTORC1 activation measured by Western blotting of a direct target of its kinase activity, T389 of p70-S6K1 (pT389-S6K). As shown in Figure 3*C*, HEK 293T cells expressing either full-length LARS or LSV3 exhibited significantly greater leucine-dependent phosphorylation at T389 of S6K compared with the empty vector control. The ratio of phosphorylated S6K increased by an average of 1.9- and 2.0-fold in LARS and LSV3 overexpressing cells, respectively, compared with control (n = 4, both *p* < 0.05 by paired *t* test). Therefore, this SV retains the noncanonical leucine sensing function of full-length LARS despite catalytic domain disruption.

Enhancement of the RagD-LARS interaction in the presence of leucine is a critical mechanistic feature of mTOR signaling (31). To determine if signaling by LSV3 uses a similar mechanism, we tested whether SV binding with RagD is dependent upon leucine. Following either leucine starvation or stimulation, HEK 293T cells expressing GST-tagged RagD in addition to LARS or LSV3 were subject to glutathione agarose pull-downs. The RagD-LSV3 interaction was clearly enhanced in the presence of leucine compared with no supplementation (Fig. 3*D*), providing further evidence that alternative splicing has preserved the noncanonical mTOR signaling activity of human LARS.

### SRSF1 RNA-binding protein is a regulator of LARS exon 20 skipping in immune cells

**Figure 4:**
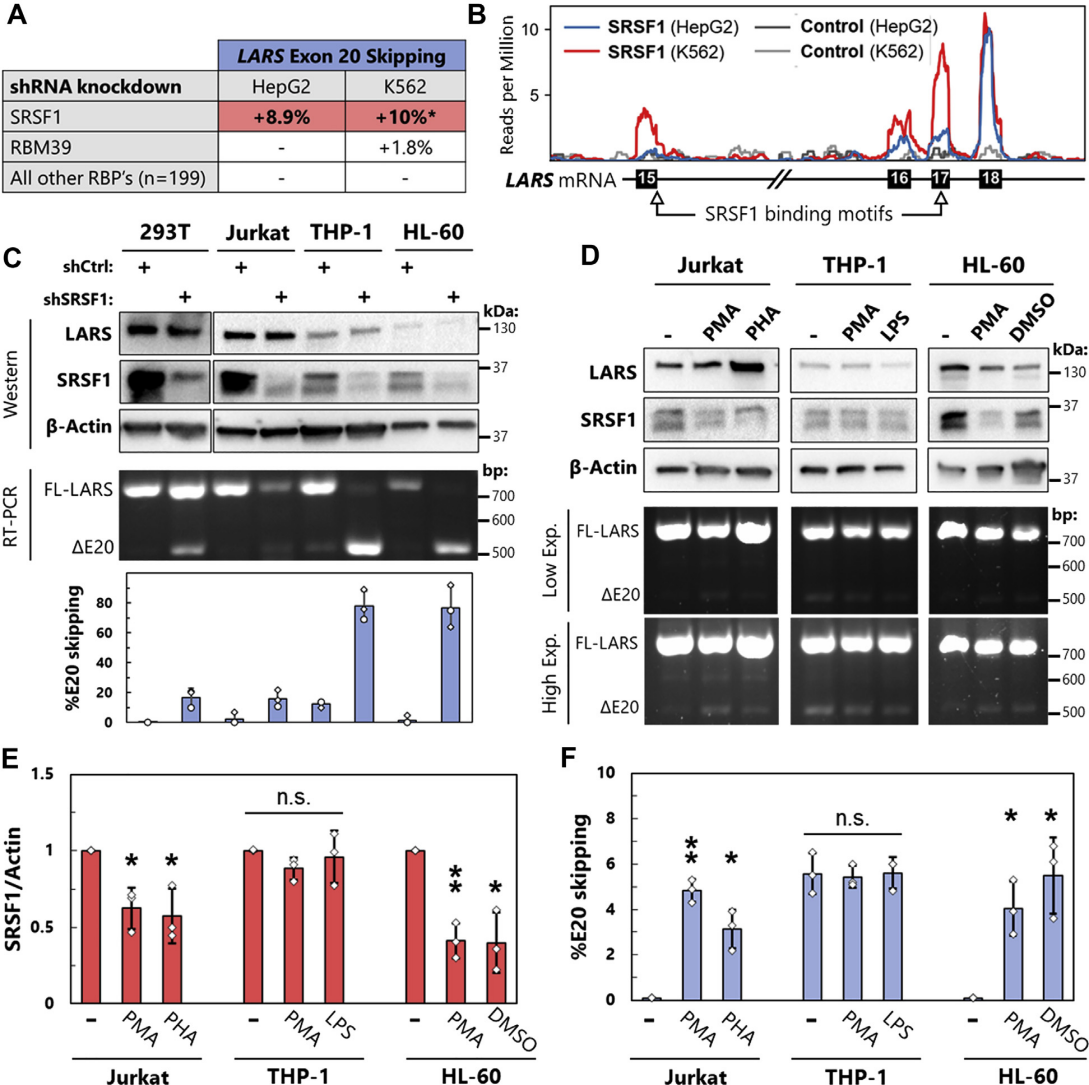
**SRSF1 regulates LSV3 expression in leukocytes.***A*, summary of data from ENCORE project, where shRNA knockdowns were performed on 201 RNA-binding proteins in HepG2 and K562 cells followed by RNAseq [26]. Alternative splicing events at *LARS* exon 20 were quantified using rMATS and are reported as changes in percent exon skipping. ∗Several additional alternative splicing events at *LARS* exon 20 not included in this % change. *B*, SRSF1 eCLIP binding data from ENCORE mapped onto *LARS* transcript. SRSF1 consensus binding motifs identified using ATtRACT database. *C*, HEK 293T epithelial cells, Jurkat T lymphocytes, THP-1 monocytes, and HL-60 promyeloblasts were treated with lentivirus containing either control or SRSF1-targeting shRNA followed by 2 days of puromycin selection. SRSF1 levels detected by Western blot, and LARS exon 20 splicing changes measured by RT-PCR. All quantifications are the result of three biological replicates. *D*, Jurkat cells were treated with phorbol 100 ng/ml phorbol 12-myristate 13-acetate (PMA) and 250 ng/ml ionomycin or 10 μg/ml of phytohemagglutinin (PHA) for 72 h, THP-1 cells with 100 ng/ml PMA for 72 h followed by 24 h with 100 ng/ml lipopolysaccharide (LPS), and HL-60 cells with 100 ng/ml PMA or 1.3% DMSO for 96 h. Agarose gel of RT-PCR products is shown at both low and high exposures (exp.). *E* and *F*, results from *D* as well as two independent biological replicates were quantified. SRSF1 protein level from western blots was normalized to β-Actin loading control, and percent *LARS* exon 20 skipping quantified from RT-PCR. n.s. = not significant, ∗*p* < 0.05, ∗∗*p* < 0.01 by paired *t* test. LARS, leucyl-tRNA synthetase.

Exon 20 skipping in LSV3 clearly disrupts the catalytic center for LARS aminoacylation, while maintaining its cell signaling function. To understand how this separation of function might occur specifically in immune tissues, and leukocytes in particular, we sought to uncover the upstream regulators of the exon 20 skipping event. We searched the ENCODE database (34, 35) for RNA-binding proteins (RBPs) that influence LARS splicing. We found that among 201 RBPs, only knockdown of serine-arginine rich splicing factor 1 (SRSF1) increased LARS exon 20 skipping in both K562 blood cells and HepG2 cells (Fig. 4*A*). The only other RBP that impacted LARS exon 20, RBM39, had a comparatively small exon skipping increase in K562 cells alone. Interestingly, knockdown of SRSF1 induced exon 20 skipping at lower levels in HepG2 liver cells than in the multipotent K562 hematopoetic cell line, where it also resulted in several cryptic splicing events at exon 20, but no other *LARS* exons (Fig. S4).

In general, SRSF1 promotes exon inclusion (36), and disrupting its natural binding to transcripts leads to skipping of nearby exons. To determine whether SRSF1 is positioned to regulate *LARS* exon 20, we searched the enhanced cross-linking immunoprecipitation (eCLIP) sequencing dataset from ENCODE (34) for SRSF1-bound transcripts. These data showed that SRSF1 interacts with the *LARS* transcript directly upstream of the skipped exon, in the region of exon 18, with greater signal detected in K562 blood cells (Fig. 4*B*). Using the ATtRACT database (37), we also identified several SRSF1 consensus binding motifs within this transcript region.

These results not only revealed SRSF1 as a potential regulator of LARS exon 20, but also suggested a cell-type-specific effect. We used the ENCODE shRNA sequence to knockdown SRSF1 in HEK 293T cells, as well as three leukocyte cell lines including Jurkat T lymphocytes and the myeloid cell lines THP-1 and HL-60. SRSF1 knockdown increased LSV3 expression as measured by RT-PCR in all cell lines tested (Fig. 4*C*). However, the two myeloid cell lines exhibited a much greater (approximately 80% exon 20 skipping) increase in SV expression than either HEK 293T or Jurkat cells, and one of these, THP-1 monocytes, was found to express some LSV3 even in the control.

SRSF1 is known as a master regulator of splicing in immune cells (38) and is downregulated during T lymphocyte activation (39). We used a range of cell culture systems to determine whether SRSF1 regulates LSV3 expression during key immunological events (Fig. 4*D*). To model T-lymphocyte activation, Jurkat cells were treated with phorbol 12-myristate 13-acetate (PMA) and phytohemagglutinin (PHA). Since myeloid cells showed particular propensity for exon 20 skipping during SRSF1 knockdown, we also used PMA to differentiate THP-1 monocytes to macrophages and treated these with bacterial lipopolysaccharide to an inflammatory macrophage phenotype. Finally, HL-60 promyeloblasts were differentiated to monocyte- and neutrophil-like states with PMA and DMSO, respectively.

As expected from the literature (38), we observed a statistically significant downregulation of SRSF1 protein upon activation of Jurkat T cells (Fig. 4*D* left panel, Fig. 4*E*). Importantly, we detected concomitant development of a small but significant amount of LARS exon 20 skipping (Fig. 4*D* high exposure, Fig. 4*F*) in these activated T cells. In contrast, THP-1 monocytes showed no significant change in SRSF1 protein during differentiation to macrophages (Fig. 4*D* middle panel, Fig. 4*E*) and accordingly maintained consistent LSV3 expression level (Fig. 4*F*). The more stem-like HL-60 promyeloblasts, however, experienced statistically significant decreases in SRSF1 protein and associated increases in exon 20 skipping upon differentiation with both PMA and DMSO (Fig. 4*D* right panel, Fig. 4, *E* and *F*). Collectively, these data show not only that LSV3 is negatively regulated by SRSF1, but also that expression of this LSV increases during specific immune cell differentiation and activation events.

## Discussion

The family of AARS are core housekeeping proteins, rooted in the very origin of the genetic code and protein synthesis. Throughout evolution, they have been adapted to be multifunctional as cells and organisms became increasingly sophisticated. We have discovered that an SV of human LARS is expressed in a highly tissue-specific manner. This exon 20 skipping event represents approximately one-fifth of *LARS* transcripts in blood leukocytes, which are comprised of a complex population of cells including lymphocytes, monocytes, neutrophils, etc. Our experiments with immune cell lines show that myeloid lineage cells are sensitive to LSV3 expression, where knockdown of SRSF1 can lead to a nearly complete switch in exon usage. Therefore, it is possible that the LSV3 transcript observed in total leukocytes may result mostly from monocytes or similar cell types with a much higher proportion of exon 20 skipping than the collective measurement of approximately 20%. This is further supported by our observations that lymphoid-dominant tissues such as thymus and spleen express significantly less LSV3 than do total leukocytes.

Immune cell lines appear to express less LSV3 compared with their primary tissue counterparts, and we were unable to detect SV protein in cultured cells. The negative regulator of exon 20 skipping that we identified, SRSF1, is known to be upregulated in many cancers (40). It is possible that increased SRSF1 in these leukemia and lymphoma cell lines may interfere with LSV3 expression that would otherwise occur in primary cells of the same type. Screening of a wide variety of primary immune tissues determined that LARS exon 20 skipping appears to be absent only in sites dominated by immature progenitor cells such as the bone marrow and fetal liver. In addition, multiple *in vitro* models of immune cell differentiation and activation show modest increases in exon 20 skipping, coinciding with decreases in SRSF1 protein. Together, these factors suggest that LSV3 expression is highest in mature immune cells, relative to their more stem-like progenitors.

The LSV3 SV contains a 71 amino acid deletion in the catalytic domain, which abolishes its canonical enzymatic function, while preserving cell signaling. The biological relevance of separating these functions is still unclear. For instance, LSV3 can sense leucine sufficiency and activate mTORC1, which in turn promotes translation. However, translation requires tRNA leucylation activity, which LSV3 lacks (21). Splicing of LARS to LSV3 could, however, conserve leucine otherwise used in aminoacylation and translation for use in catabolism. This strategy is notably employed during glucose starvation, in which LARS is phosphorylated to inhibit both its catalytic and cell signaling activities and conserve energy (24). Regulation by splicing to LSV3 would have the advantage of preserving mTOR activity while still disrupting aminoacylation and therefore sparing leucine. This could be especially important in immune cells, which require mTOR signaling for energy-demanding processes such as T-cell activation and macrophage development (41, 42).

Many alternative functions of AARS are integral to immune system functioning (6, 8, 14, 17). What consequence might splicing of LARS to LSV3 have for leukocytes in particular? Interestingly, high concentrations of branched chain amino acids, particularly leucine, have been linked to inflammation and cardiovascular disease because of their potency in activating mTOR in leukocytes (43, 44, 45). It is possible that this strong mTOR response in leukocytes results from the tissue-specific expression of the specialized leucine sensor LSV3. Furthermore, the regulator of LARS exon 20 skipping, SRSF1, is a key modulator of immune biology, controlling the splicing of several key cytokines and cell surface receptors (38, 39). Reduced SRSF1 expression in T cells is correlated with autoimmune disease, hypersensitivity, and inflammation (46), and these phenotypes result in part from elevated mTOR activity (47). We hypothesize that low SRSF1 levels in disease contexts would lead to increased LSV3 expression and in turn contribute to the aberrantly high mTOR activity associated with these inflammatory symptoms.

The plethora of noncanonical functions of tRNA synthetases (3) support that they are widely embedded throughout biology. How AARS balance their roles in translation with other unrelated, yet still critical, responsibilities is often unclear. Regulation upstream of the alternative function, for example, must be specific or subtle enough to not entirely disrupt the essential catalytic function. Here we have shown how alternative splicing can be used to separate evolutionarily ancient aminoacylation from a more recent cell signaling role, creating a functionally specified protein distinct from the moonlighting LARS. With the knowledge that hundreds of other AARS SVs exist in humans (20), we anticipate that this is only one of many examples of how these ancient proteins can be adapted for new roles in eukaryotes using alternative splicing.

## Experimental procedures

### Materials

The pCDNA3-FLAG-LARS and pVITRO-myc-hVps34 vectors were provided by Professor J. Chen at the University of Illinois Urbana-Champaign (UIUC) and were previously described (22). To generate the FLAG-LSV3 vector, FLAG-LARS was PCR amplified in two fragments omitting exon 20, and these were joined by Gibson assembly (New England Biolabs #E2611). The FLAG-LARS and -LSV3 coding sequences were subcloned into pET-19b using the NdeI and XhoI restriction sites to create p19-LARS and p19-LSV3. For shRNA knockdowns, pLKO.1-scramble (shCtrl), pCMV-VSV-G and pCMV-ΔR-8.2 vectors were provided by Professor L.F. Chen at UIUC (48), while pLKO.1-shSRSF1 (TRCN0000001095) was purchased from Sigma-Aldrich. All other vectors were obtained *via* Addgene: FLAG-HA-pcDNA3.1 (EV, #52535) from A. Antebi (49), pRK5-HA-GST-RagD (GST-RagD, #19307) from D. Sabatini (32), and HA-EglN2-pCDNA3 (HA-PHD1, #18961) from W. Kaelin (50).

The HEK 293T cells were provided by Professor D.J. Shapiro at UIUC and were maintained in DMEM with 10% fetal bovine serum (FBS), 100 units/ml penicillin, and 100 μg/ml streptomycin (pen-strep). Jurkat T-lymphocytes were received from Professor L.F. Chen (UIUC), while THP-1 monocytes (TIB-202) and HL-60 promyeloblasts (CCL-240) were both purchased from the American Type Culture Collection (ATCC) and were all maintained in RPMI-1640 with 10% FBS, pen-strep, 10 mM HEPES, 2 mM L-glutamine, and, in the case of THP-1 cells, supplemented with 0.05 mM 2-mercaptoethanol. All mammalian cell culture media used were prepared by the UIUC School of Chemical Sciences Cell Media Facility. Radioactive [^3^H]-leucine (#NET116600) and [^32^P]-PP_*i*_ (#NEX01900) were from PerkinElmer. Antibodies used are listed in Table 1.

**Table 1:** Antibodies used for Western blotting and immunoprecipitation

Antigen	Species	Source	Catalog #	Dilution
FLAG-tag	Mouse	Sigma-Aldrich	F1804	1:4000
LARS	Rabbit	Zhang & Martinis^a^	-	1:4000
β-actin	Rabbit	Cell Signaling Technologies	13E5	1:1000
EPRS	Rabbit	Abcam	ab31531	1:20,000
IARS	Rabbit	Abcam	ab31533	1:2000
Vps34	Rabbit	Echelon Biosciences	Z-R015	1:1000
Myc-tag	Mouse	Cell Signaling Technologies	2276	1:1000
pT389-S6K	Rabbit	Cell Signaling Technologies	9205	1:500
GST	Rabbit	Abcam	ab9085	1:10,000
HA-tag	Rabbit	Sigma-Aldrich	H6908	1:1000
SRSF1	Mouse	Thermo Fisher	32-4500	1:1000
Rabbit IgG (HRP conjugate)	Goat	Seracare (KPL)	074-1406	1:10,000
Mouse IgG (HRP conjugate)	Goat	Seracare (KPL)	074-1806	1:10,000

a Manuscript in preparation. Briefly, for production of the rabbit anti-LARS antibody used here, purified recombinant LARS protein was submitted to Pierce Custom Services (Thermo Fisher). Antibodies were purified from rabbit serum provided by this service using cyanogen bromide activated-sepharose resin (Sigma, C9142) coupled to LARS protein.

### Pacific Biosciences sequencing

A primer to the 3′ end of the *LARS* coding sequence with an appended SMART CDS site (5′-AAGCAGTGGTATCAACGCAGAGTACTGTGCATGAGTTTAATGAAC-3′), was used to generate a cDNA library from polyA-selected leukocyte RNA (Takara #636170) using the SMARTer PCR cDNA synthesis kit (Takara #634925). The National Center for Genome Resources (NCGR, Santa Fe, NM) completed construction of the cDNA library, using the SMARTer kit’s 5′ PCR Primer II A, followed by size selection and sequencing on two cells of a Pacific Biosciences (PacBio) Sequel II system. Raw data were processed by NCGR using the PacBio IsoSeq pipeline (25) to generate consensus isoforms and remove short reads and chimeras. Reads were then aligned to the *LARS* gene sequence (NCBI accession NM_020117.10) with Clustal Omega.

To reduce errors and sequencing artifacts that would interfere with detection of exon deletions, the PacBio sequencing dataset was filtered to aligned transcripts where:

$$\ln\left(\frac{AlignedBases}{CigarInstructions}\right)\geq 4$$

This eliminated alignments interrupted by numerous small insertions or deletions. Each gap in a read from the filtered alignment dataset was mapped to the nearest exon boundaries and scored according to:

$${\left(1+\left|Start-NearExonStart\right|+\left|End-NearExonEnd\right|\right)}^{-1/2}$$

Skipping events with total scores below 1 were discarded. For quantification, a read was determined to have an exon skipping event if at least 80% of positions inside it had no aligned base and at least half the positions in the margins around the deletion had an aligned base, where each margin was 20% the length of the potential deletion.

To detect partial exon-skipping events (alternative 3′ or 5′ splice sites), the filtered alignment dataset was searched for deletions appearing in at least four alignments, as suggested by the “gap quality score” (51), with one endpoint not at an exon boundary. For quantification, collections of deletions were merged with any deletions with respective endpoints at most three bases away and deletions were allowed to bridge small “islands” of at most three bases in the middle of a gap, accounting for sequencing or alignment errors. Partial intron retention events from ENCODE Project data were searched for and counted manually.

### Recombinant protein purification

Rosetta 2 DE3 (Novagen) *E. coli* containing p19-LARS or p19-LSV3 was grown overnight in LB at 37 °C with 100 μg/ml ampicillin and 50 μg/ml chloramphenicol to an OD_600_ of approximately 0.5 and then cooled on ice for 1 h followed by induction with 0.5 mM isopropyl β-D-1-thiogalactopyranoside (IPTG) and 16 h of vigorous shaking at room temperature. The six-His-tagged recombinant proteins were purified using nickel-affinity chromatography with HisPur Ni-NTA resin (Thermo Fisher Scientific #88221), followed by a Superdex 200 HiLoad 16/600 (GE Life Sciences) size-exclusion column in running buffer (25 mM HEPES pH 7.5, 150 mM NaCl, 5% glycerol) and subsequent storage at −20 °C in running buffer supplemented with 50% glycerol.

### Enzyme activity assays

To increase aminoacylation efficiency, bovine liver total tRNA (Sigma #R4752) was treated with recombinant *E. coli* CCA-adding enzyme (52) in a reaction mix containing 100 mM Tris pH 8.1, 10 mM MgCl_2_, 1 mM dithiothreitol (DTT), 5 mM each ATP and CTP, 5 μM enzyme, and 1.6 mg/ml of crude tRNA. Reactions were incubated at 37 °C for 15 min, followed by phenol-chloroform extraction, ethanol precipitation, and storage in 10 mM HEPES pH 7.5 and 0.1 mM EDTA.

The tRNA was refolded by incubating with 2.5 mM MgCl_2_ at 95 °C for 1 min, followed by 30 min on ice. For measurement of tRNA leucylation activity, reactions were carried out in 50 mM HEPES, pH 7.4, 25 mM KCl, 10 mM MgCl_2_, 1 mM DTT, 4 mM ATP, 2 mg/ml CCA-treated tRNA, 50 nM recombinant LARS or LSV3, and 40 μM [^3^H]-leucine (10 μCi/nmol). Reactions were incubated at 37 °C, with 5 μl aliquots removed and quenched on 10% trichloroacetic acid (TCA) soaked filter paper. Filter paper pads were washed by slowly shaking for 30 min in 10% TCA once, followed by two washes in 5% TCA and one in 95% ethanol. Dried pads were then placed into 4 ml toluene PPO scintillation fluid (Fisher #T313-4), and radioactivity was measured *via* a Beckman LS 6500 scintillation counter.

Pyrophosphate exchange assays were conducted in 50 mM HEPES, pH 7.4, 10 mM MgCl_2_, 1 mM DTT, 1 mM leucine, 4 mM ATP, 50 nM recombinant LARS or LSV3, and 2 mM of [^32^P]-PP_*i*_ (2.5 mCi/mmol). At various time points, 5 μl was spotted onto PEI-cellulose TLC plates (Macheery-Nagel #801063) that had been prerun in deionized water. Nucleotides were separated in 750 mM KH_2_PO_4_ pH 3.5 with 4 M urea. Radioactive TLC plates were exposed to phosphorimager screens, imaged on a Molecular Dynamics Storm 840, and quantified using the manufacturer software.

### Human cell culture

Transfection of HEK 293T cells was performed using Lipofectamine 2000 (Thermo Fisher #11668019) and the indicated vectors for 48 h. For measurements of mTOR activity and LARS-RagD interaction leucine sensitivity, HEK 293T cells were grown in poly-D-lysine (Sigma #P6407) treated plates to prevent detachment and were transfected with the indicated DNA for 24 h, followed by 18 to 20 h in serum-free DMEM. Cells were washed once in PBS followed by 2 h starvation in leucine-free DMEM without serum and then 20 to 30 min stimulation with 4 to 8 mM leucine.

To prepare shRNA-containing lentivirus for SRSF1 knockdowns, HEK 293T cells were transfected with pLKO.1-scramble or pLKO.1-shSRSF1 as well as pCMV-VSV-G and pCMV-ΔR-8.2. Lentivirus containing media was collected over the next 2 days and concentrated *via* ultracentrifugation at 100,000*g* for 90 min and the virus resuspended in PBS. For knockdowns, 1 × 10^6^ cells were treated with 2 μl of concentrated virus (MOI ∼100) for 1 day and selected in varying concentrations of puromycin for a further 2 days before analysis.

For immune cell line differentiation and activation experiments, Jurkat cells at 2 × 10^5^ cells/ml were treated with 100 ng/ml of phorbol 12-myristate 13-acetate (PMA) and 250 ng/ml of ionomycin (Sigma #P8139 and #I9657) or 10 μg/ml of phytohemagglutinin-L (PHA, Sigma # 11249738001) for 72 h. THP-1 cells at 3 × 10^5^ cells/ml were treated with 100 ng/ml PMA for 72 h, followed by 24 h with 100 ng/ml of *E. coli* O111:B4 lipopolysaccharide (Sigma #L2630). HL-60 cells at 3 × 10^5^ cells/ml were treated with either 1.3% v/v DMSO or 100 ng/ml of PMA for 96 h.

### Immunoprecipitations and Western blotting

For FLAG-tag IP, HEK 293T cells transfected with FLAG-LARS or LSV3 were lysed in 25 mM Tris pH 7.5, 10 mM NaCl, 2 mM EDTA, 1% NP-40, 1 mM phenylmethylsulfonyl fluoride (PMSF), and 1% cOmplete EDTA-free protease inhibitor cocktail (Roche #11836170001) by shaking at 4C for 1 h Five percent of lysate was collected as input samples and the remainder incubated at 4 °C with FLAG-M2 affinity resin (Sigma #A2220) for 6 h. Resin was pelleted by gentle centrifugation and washed three times in lysis buffer supplemented to 100 mM NaCl. Immunoprecipitates were eluted *via* boiling in 2× Laemmli buffer (Bio-Rad #1610737EDU) with 5% 2-mercaptoethanol.

Pierce glutathione agarose (Thermo Scientific #16101) was used for GST-RagD pull-downs, and samples were treated as above except for the addition of 5 mM DTT to lysis and wash buffers. For Myc-Vps34 IP, cells were lysed in MIPT buffer (25 mM Tris pH 7.5, 25 mM NaF, 25 mM β-glycerophosphate, 0.1 mM Na_2_VO_4_, 2 mM EDTA, 1 mM DTT, 0.3% Triton X-100, and 0.5 mM PMSF). Lysates were incubated with 0.5 μg of either control rabbit IgG (Cell Signaling Technology #2729) or anti-Myc overnight. Protein A magnetic beads (Bio-Rad #161-4811) were added to lysates and rotated at 4 °C for 1 h, followed by three washes in MIPT supplemented with 50 mM NaCl and boiling in Laemmli buffer to elute.

For general blotting analysis, cells were lysed in 40 mM HEPES pH 7.4 with 1% Triton X-100, 10 mM β-glycerophosphate, 10 mM pyrophosphate, 2.5 mM MgCl_2_, and 1% cOmplete protease inhibitor by gently shaking at 4 °C for 30 min, followed by 10 min of centrifugation at 15,900*g* and 4 °C. Clarified lysate was mixed with 2× Laemmli sample buffer, separated *via* PAGE, transferred to PVDF membranes (Bio-Rad #1620177), and membranes blocked in PBS with 0.05% Tween 20 (PBST) and 4% bovine serum albumin (BSA, Sigma #A7906). Membranes were then treated with the indicated primary and secondary antibodies in PBST with 4% BSA and developed in SuperSignal West Femto substrate (Thermo Scientific #34094) before imaging. Bands were quantified using ImageLab software (Bio-Rad).

### RNA extraction and RT-PCR

RNA was extracted from cultured cell lines using the Omega Biotek EZRNA kit (#R6812-02), followed by treatment with 2 units of New England BioLabs (NEB) RNase-free DNase I (#M0303). An aliquot of 0.5 to 1 μg of RNA was used for reverse transcription with the Bio-Rad iScript cDNA synthesis kit (#1708891). For screening of multiple tissues, cDNAs from the human immune system MTC panel and human MTC panel 1 from Takara (#636748 and #636742) were used as template. To detect *LARS* exon 20 skipping in all cases, PCR was performed using NEB OneTaq DNA polymerase (#M0482) and LSV3-F (5′- GCTCTGTGTGACCAGTGGTACT-3′) and LSV3-R (5′-GAATACCTGCATCTGCCATGGC-3′) primers. Amplification products were analyzed *via* gel electrophoresis in 1.5% agarose with ethidium bromide staining, and bands were quantified as above.

### ENCODE database analyses

To identify proteins controlling *LARS* splicing, the ENCODE Project database (34, 35) was searched for “shRNA RNA-seq” experiments knocking down specific proteins in human cell lines HepG2 and K562. Accession numbers are listed in Table S1 in supporting information. Each rMATS “differential splicing quantifications” output file was downloaded. All skipped-exon (SE) entries affecting *LARS* splicing with *p* < 0.01 were saved and tagged with the source experiment’s cell line and target protein. Start and end base positions were mapped to exons using the hg19 assembly annotations table.

To determine whether SRSF1 binds to the *LARS* transcript, the ENCODE Project database was searched for “eCLIP” (enhanced cross-linking immunoprecipitation) experiments on SRSF1. For each cell line, the merge_peaks output file based on the hg19 assembly was downloaded. Experiment and accession numbers are listed in Table S2 in supporting information. The ATtRACT database [28] was used to locate instances of the SRSF1-binding motif on the primary *LARS* transcript near the regions identified in the eCLIP results.

## Data availability

The data used to support the findings of this study are available from the corresponding author upon request.

## Supporting information

This article contains supporting information (22, 28).

## Conflict of interest

The authors declare that they have no conflicts of interest with the contents of this article.
