# Supplementary material for: Tissue-specific alternative splicing separates the catalytic and cell signaling functions of human leucyl-tRNA synthetase
Source: J Biol Chem. 2022 Feb 21;298(4):101757. doi: 10.1016/j.jbc.2022.101757 (PMC8941210; doi:10.1016/j.jbc.2022.101757)
Supplement: Supplemental Figures S1–S4 [file mmc2.pdf]

Supporting Information:

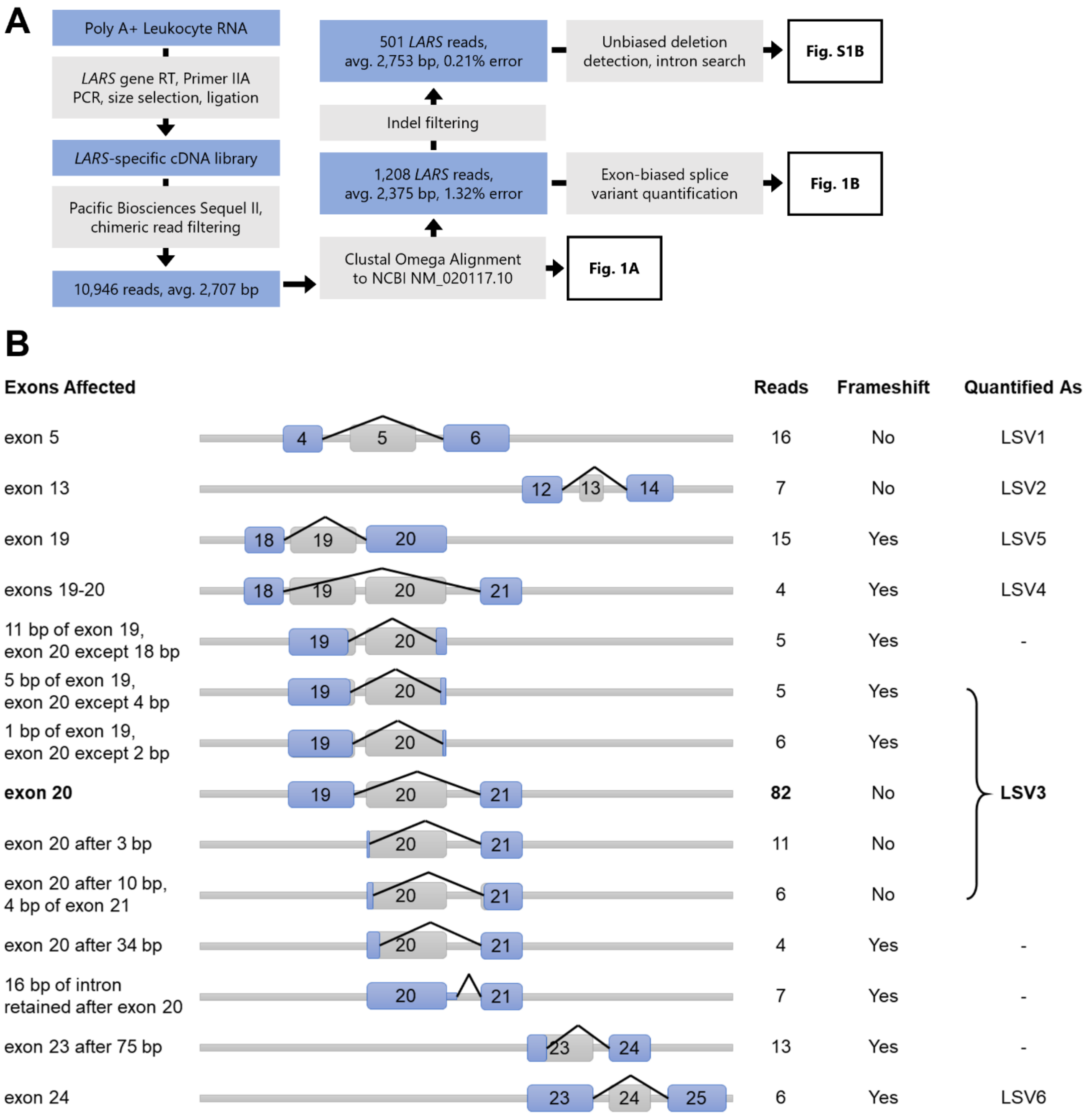

Figure S1: Pacific Biosciences sequencing of *LARS* in leukocytes

A. Workflow for *LARS* gene-specific mRNA isoform sequencing using Pacific Biosciences, showing downstream analysis steps as well as the datasets and figures generated from each of them. **B.** Line diagrams of all transcript isoforms defined during unbiased (no exon information) deletion and insertion search from highest quality filtered PacBio data. To allow for the high error rate and indel frequency with this sequencing method, isoforms with small errors around the skipped exon were permitted in the quantification (see Fig. 1B and methods).

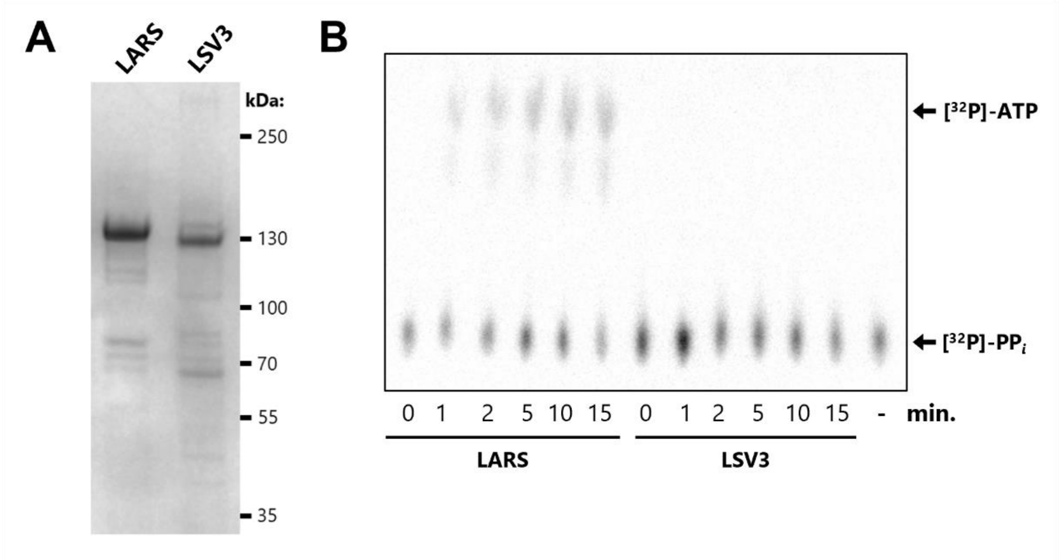

Figure S2: Purification and enzymatic assays of LSV3 protein

A. Coomassie-stained polyacrylamide gel of eletrophoretically separated purified recombinant LARS and LSV3 proteins used in enzymatic assays. **B.** Example of thin layer chromatography plate of pyrophosphate exchange reactions quantified in Fig. 2E. Radiolabeled ATP and PP_i were separated using PEI-cellulose plates run in 750 mM KH_2PO_4 pH 3.5 with 4 M urea.

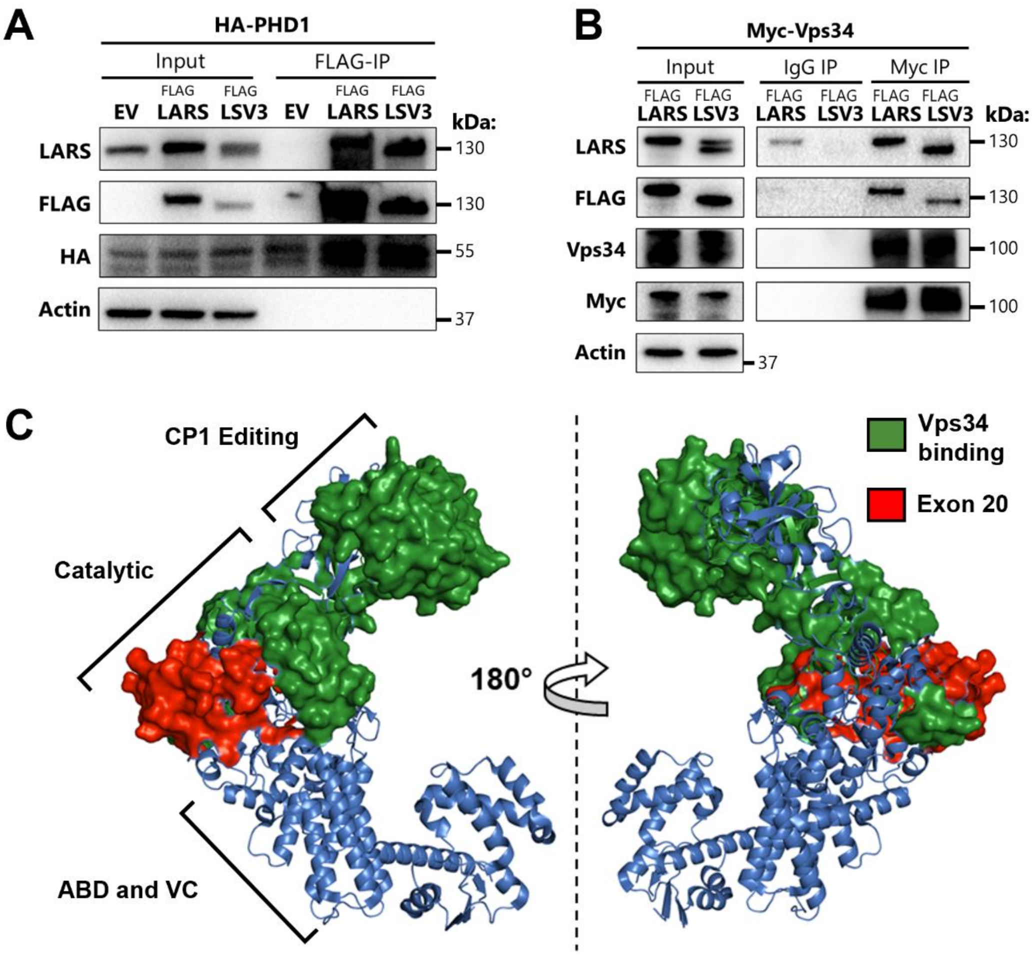

Figure S3: LSV3 interacts with PHD1 and Vps34 signaling partners

A. Co-immunoprecipitation (co-IP) of FLAG-tagged LARS and LSV3 from HEK 293T cells overexpressing HA-tagged EglN2, also known as prolyl-hydroxylase domain-containing protein 1 (PHD1). **B.** Co-IP of Myc-Vps34 from HEK 293T cells overexpressing FLAG-tagged LARS and LSV3, including untargeted IgG control. **C.** Structure of human LARS (PDB 6LPF) (28) showing region with implicated in Vps34 binding in green (22), while peptide encoded by exon 20 is shown in red.

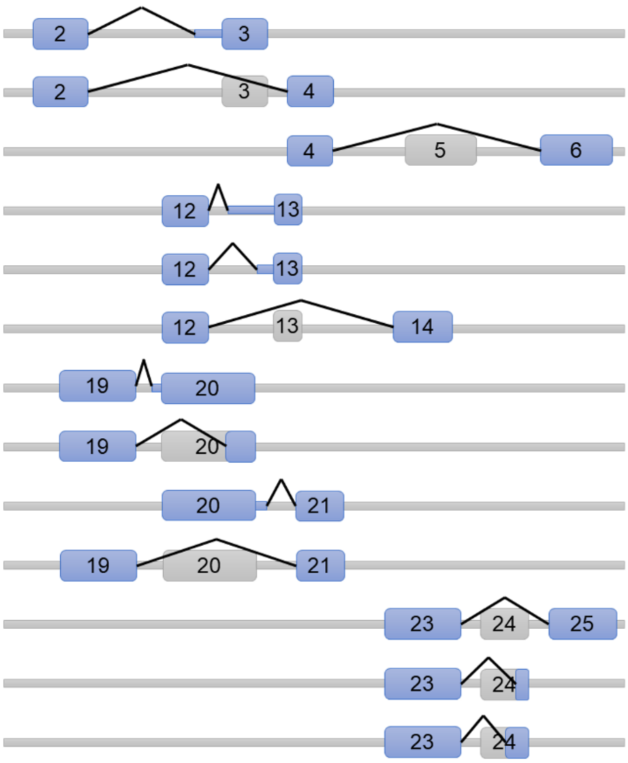

Name	Exons Affected	Frameshift	RBPs with splicing changes In K562	RBPs with splicing changes In HepG2
-	52 bp then exon 3	Yes	AQR	-
-	exon 3	Yes	-	**SRSF1**
LSV1	exon 5	No	RAVER1, SF3B4	U2AF2
-	91 bp then exon 13	Yes	AQR	-
-	25 bp then exon 13	Yes	AQR	-
LSV2	exon 13	No	(49)	(3)
-	21 bp then **exon 20**	No	**SRSF1**	-
-	first 143 bp of **exon 20**	Yes	**SRSF1**	-
-	**exon 20** then 16 bp	Yes	**SRSF1**	-
**LSV3**	**exon 20**	No	RBM39, **SRSF1**	**SRSF1**
LSV6	exon 24	Yes	(20)	PABPC1, U2AF1
-	first 29 bp of exon 24	Yes	MAGOH	-
-	first 46 bp of exon 24	No	EIF4A3	-

Figure S4: *LARS* transcripts present in the ENCODE dataset

Line diagrams of all *LARS* alternatively spliced transcript isoforms detected in the search of the ENCODE database's RBP knockdown followed by RNA-seq experiments. Exons 13 and 24 (LSV2 and LSV6) underwent splicing changes upon knockdown of many RBPs. SRSF1 is the only RBP whose decrease led to exon 20 splicing changes in both tested cell lines.
